# Stoichiometric Homeostasis and Functional Group Divergence Jointly Enhance Alpine Plant Adaptation to Environmental Stress

**DOI:** 10.3390/plants14182835

**Published:** 2025-09-11

**Authors:** Aihui Ma, Zhe Chen, Xin Jing, Yu Chen, Jinhong Guan, Shixiong Wang, Wenying Wang, Huakun Zhou, Jian Sun, Xufeng Mao, Yanxia Jin

**Affiliations:** 1 Key Laboratory of Tibetan Plateau Land Surface Processes and Ecological Conservation (Ministry of Education), Qinghai Normal University, Xining 810016, China; maaihui01@outlook.com (A.M.); chenyu258x@163.com (Y.C.); cocogjh@163.com (J.G.); wangshix812@163.com (S.W.); wangwy0106@163.com (W.W.); maoxufeng@yeah.net (X.M.); jinyanx526@163.com (Y.J.); 2 Academy of Plateau Science and Sustainability, Xining 810016, China; 3 Qinghai Key Laboratory of Biodiversity Formation Mechanism and Comprehensive Utilization of the Qinghai-Tibetan Plateau, Xining 810016, China; 4 State Key Laboratory of Herbage Improvement and Grassland Agro-Ecosystems, College of Pastoral Agriculture Science and Technology, Lanzhou University, Lanzhou 730020, China; jingx@lzu.edu.cn; 5 Qinghai Key Laboratory of Restoration Ecology of Cold Area, Northwest Institute of Plateau Biology, Chinese Academy of Sciences, Xining 810008, China; hkzhou@nwipb.cas.cn; 6 State Key Laboratory of Earth System Resources and Environment of Tibetan Plateau, Institute of Tibetan Plateau Research, Chinese Academy of Sciences, Beijing 100101, China; sunjian@itpcas.ac.cn

**Keywords:** Tibetan Plateau, alpine grassland, altitudinal gradient, plant functional group, stoichiometric homeostasis

## Abstract

Climate warming promotes alpine plant migration to higher elevations, yet how they adapt via stoichiometric homeostasis remains unclear. We measured plant C, N, and P traits and homeostasis across community and functional group levels in three alpine vegetation types—meadow (3200–3400 m), shrubland (3400–3700 m), and cushion vegetation (3700–4400 m)—along an altitudinal gradient in the northern Qilian Mountains, Tibetan Plateau. Shrubland, as ecotones, had higher soil C and N, with plant communities showing the highest N and N:P but lowest C:N. At the functional group level, Poaceae (Gramineae) and forbs had the highest N and N:P, while Cyperaceae had the highest P in shrubland. Notably, Cyperaceae in shrubland exhibited weak P and C:P homeostasis. Poaceae (Gramineae) were mainly influenced by soil, Cyperaceae by climate, and forbs by elevation. Structural equation modeling showed elevation indirectly affected stoichiometry via climate and soil; climate influenced nutrient contents, while soil controlled C:N:P ratios. These results reveal diverse nutrient regulation and survival strategies in alpine plants, enhancing understanding of adaptation and community assembly under climate change.

## 1. Introduction

Ecological stoichiometry is the study of the balance of multiple chemical elements in ecological systems [1]. By analyzing how elements accumulate and transfer between organisms and their environment, ecological stoichiometry links elemental composition to ecosystem functions and processes [2], offering a powerful tool to understanding the relationships among organismal life strategies, ecosystem structure and function, and environmental conditions [3]. Among all essential elements, carbon (C), nitrogen (N), and phosphorus (P) are the most critical nutrients for plant growth and physiological functioning [4]. Plant stoichiometric traits are primarily determined by the availability of nutrients in the environment. For example, in alpine treeline ecosystems across seven temperate regions globally, leaf N and P contents are positively correlated with soil N and P availability [5]. Additionally, plants can adjust their internal elemental composition to cope with environmental changes. In Arctic and subarctic tundra ecosystems, significant elevational differences in microbial C, N, and P contents have been observed between shrubland and meadow communities [6]. Notably, N and P cycling respond differently to elevation depending on vegetation type: net N mineralization in shrubland soils is generally unaffected by elevation, whereas meadow soils exhibit increasing mineralization rates with elevation [6]. Therefore, understanding the stoichiometric characteristics of C, N, and P in plant–soil systems is critical for revealing how organisms respond to environmental variation and adjust nutrient use strategies to cope with change.

Nitrogen and phosphorus limitations are widespread in Arctic tundra and alpine ecosystems globally [7,8,9,10], shaping distinct stoichiometric patterns in plant nutrient allocation. For instance, low nutrient availability in soils often results in reduced leaf N and P content and elevated C:N or C:P ratios in plants [11,12,13]. Even within a single plant, stoichiometric traits vary depending on soil nutrient availability [14]. Nutrient addition experiments have shown that increasing soil N and P availability can rapidly alter plant elemental content and stoichiometric ratios (e.g., C:N, N:P), potentially leading to stoichiometric niche homogenization among coexisting plants and decreasing plant diversity [15]. Thus, both under natural and anthropogenically altered nutrient conditions, the supply of soil nutrients profoundly influences plant stoichiometric traits and affects community structure by mediating interspecific differences in nutrient composition and resource acquisition strategies.

In alpine ecosystems, soil nutrients are key drivers of plant stoichiometry. However, whether elevation indirectly regulates stoichiometric patterns by influencing material and energy redistribution and soil development, nutrient cycling remains a critical scientific question. Plant functional groups—plant groups that respond similarly to environmental gradients due to shared morphological, physiological, or life history traits [16]—are often used to reveal plant–environment relationships. In mid- and low-latitude mountains with clear vegetation zonation from forest to alpine cushion plant communities, plant turnover and functional group replacement are evident [6,17]. Different functional groups exhibit distinct ecological adaptations shaped by environmental factors such as water and temperature. For example, Poaceae (Gramineae) plants typically exhibit high N uptake efficiency and adopt a competitive strategy through rapid growth, while Cyperaceae plants require fewer nutrients and are tolerant of harsh, nutrient-poor conditions, showing a stress-tolerant strategy [18]. In ecotones, the structure and composition of functional groups are also crucial to ecological functions. In China’s forest–steppe transition zone, for instance, high biodiversity and ecosystem stability are observed. When the soil C:N:P ratio decreases, microbial investment shifts from nutrient-acquiring enzymes to C-acquiring enzymes [19]. We hypothesize that the stoichiometric differentiation of plant functional groups along elevational gradients is related to their resource acquisition strategies, which are likely ecosystem specific.

Stoichiometric homeostasis refers to the ability of organisms to maintain relatively constant internal nutrient ratios despite environmental fluctuations [1]. Plants with high homeostasis tend to have higher dominance and stability and adopt conservative nutrient-use strategies [20,21,22]. Conversely, under conditions of high resource variability, some plants may adopt more opportunistic nutrient-use strategies. In the alpine meadow of the Tibetan Plateau, *Carex parvula*, a dominant plant, shows relatively stable leaf C:N:P ratios across water–temperature gradients, indicating strong stoichiometric homeostasis and a conservative resource-use strategy [23]. Similarly, in Antarctic tundra ecosystems, slow-growing cryptogams like lichens and mosses also exhibit strong stoichiometric homeostasis, enabling survival in extremely cold, UV-intense, and nutrient-poor environments [24]. These findings suggest that alpine plants are generally stress-tolerant and nutrient-conservative. However, the upward shift of vegetation zones in recent decades implies that high-elevation plants are not static, and responsive plants or functional groups may drive treeline and grassline upward movement [25]. We therefore further hypothesize that, in heterogeneous and nutrient-limited alpine environments, functional groups adopt diverse nutrient accumulation strategies, and stoichiometric homeostasis is not always absolute.

Alpine grassland ecosystems represent some of the most stressful environments for plant growth, characterized by strong radiation, low temperatures, and nutrient poverty. These stressors lead to plant compositions and community structures distinct from those in temperate or tropical systems. Moreover, alpine grassland ecosystems are highly sensitive to global climate change, and any environmental shift may significantly affect plant growth and community dynamics. Studying the stoichiometric traits of alpine grassland vegetation along elevational gradients can reveal potential plant adaptation mechanisms to climate warming. This study focuses on the northern Tibetan Plateau (Qilian Mountains), encompassing three vegetation types: alpine meadow, alpine shrubland, and alpine cushion vegetation. By examining plant stoichiometry and homeostasis at both the community and functional group levels, we aim to explore the relationships among elevation, climate, and elemental traits. In our view, alpine shrubland ecosystems, as ecotones between meadow and cushion vegetation, exhibit distinct stoichiometric patterns and nutrient-use strategies. Specifically, Cyperaceae, which are more water-dependent, and Poaceae (Gramineae), which are N-demanding, may show stronger stoichiometric homeostasis, whereas forbs may adopt a more opportunistic strategy and exhibit weaker homeostasis.

## 2. Results

### 2.1. Soil Stoichiometric Characteristics

**Figure 1:**
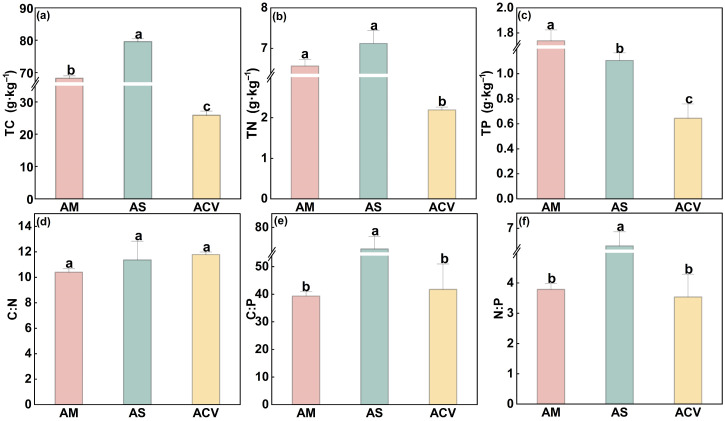
Soil total carbon (TC), total nitrogen (TN), total phosphorus (TP) contents, and their stoichiometric ratios (C:N, C:P, N:P) under different vegetation types. AM, alpine meadow; AS, alpine shrubland; ACV, alpine cushion vegetation. (**a**), C content; (**b**), N content; (**c**) P content; (**d**), C:N ratio; (**e**), C:P ratio; (**f**), N:P ratio. Different lowercase letters indicate significant differences among vegetation types (*p* < 0.05).

Soil total C (TC), as well as the soil C:P and N:P ratios, were significantly (*p* < 0.05) higher in the alpine shrubland (79.55 g·kg^−1^, 72.21, and 6.42, respectively; Figure 1a,e,f) than in the alpine meadow and alpine cushion vegetation. The lowest total N (TN) was observed in the alpine cushion vegetation (2.19 g·kg^−1^), approximately one-third of the values in meadow and shrubland soils. Soil total P (TP) showed a decreasing trend across the three vegetation types (Figure 1c), with the highest in alpine meadow (1.74 g·kg^−1^), followed by shrubland (1.11 g·kg^−1^), and the lowest in cushion vegetation (0.64 g·kg^−1^).

### 2.2. Plant Stoichiometric Characteristics

**Figure 2:**
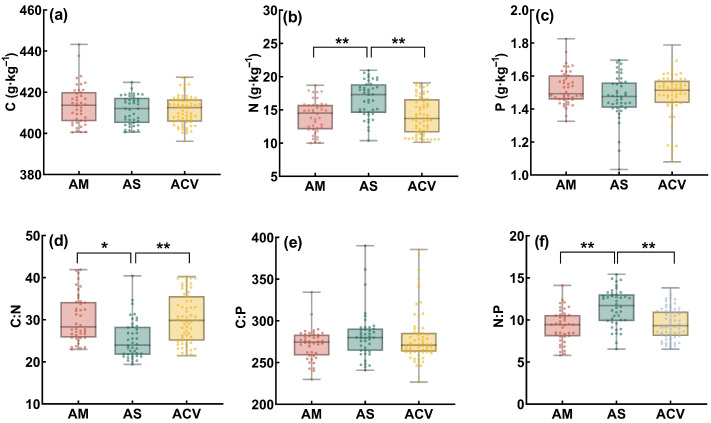
Plant carbon (C), nitrogen (N), phosphorus (P) contents, and their stoichiometric ratios (C:N, C:P, N:P) under different vegetation types. AM, alpine meadow; AS, alpine shrubland; ACV, alpine cushion vegetation. (**a**), C content; (**b**), N content; (**c**) P content; (**d**), C:N ratio; (**e**), C:P ratio; (**f**), N:P ratio. * indicates *p* < 0.05; ** indicates *p* < 0.01.

At the community level, the N and the N:P ratio in alpine shrubland plants (16.69 g·kg^−1^ and 11.4, respectively) were significantly higher than those in alpine meadow (14.06 g·kg^−1^, 9.29) and alpine cushion vegetation (14.07 g·kg^−1^, 9.53), while the C:N ratio was the lowest (26.85; Figure 2b,d,f). Notably, although there was a substantial elevational difference between meadow and cushion vegetation, and N in cushion vegetation was only about 30% of that in meadow soil (Figure 1b), their plant community-level N, C:N, and N:P values were not significantly different.

**Figure 3:**
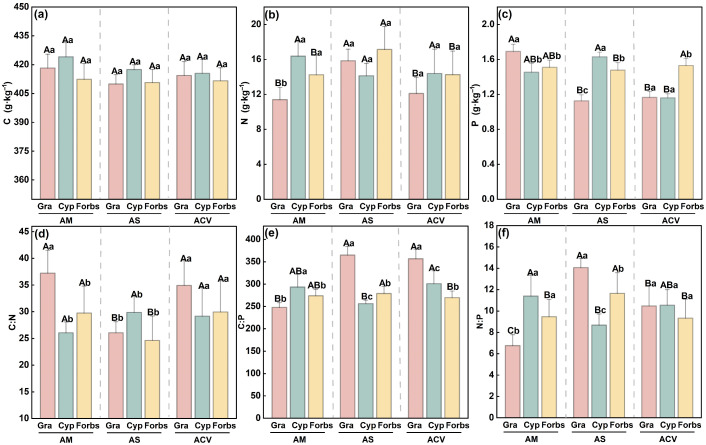
Carbon (C), nitrogen (N), phosphorus (P) contents, and stoichiometric ratios (C:N, C:P, N:P) of different plant functional groups under various vegetation types. AM, alpine meadow; AS, alpine shrubland; ACV, alpine cushion vegetation; Gra, gramineae; Cyp, cyperaceae; (**a**), C content; (**b**), N content; (**c**) P content; (**d**), C:N ratio; (**e**), C:P ratio; (**f**), N:P ratio. Different lowercase letters indicate significant differences among functional groups within the same vegetation type; different uppercase letters indicate significant differences in the same functional group across vegetation types (*p* < 0.05).

At the functional group level, in the alpine shrubland, both Gramineae and forbs had significantly higher N (N: 15.83 g·kg^−1^ and 17.15 g·kg^−1^, respectively) and N:P ratios (14.06 and 11.65), while exhibiting the lowest C:N ratios (26.06 and 24.63; Figure 3b,d,f), compared to the other two vegetation types. In contrast, stoichiometric traits of Cyperaceae were more closely associated with phosphorus content and related ratios. Specifically, P content and C:P, N:P ratios differed significantly across vegetation types. Notably, the lowest C:P (256.33) and N:P (8.68) ratios were observed in alpine shrubland, whereas TP content was highest in shrubland (1.63 g·kg^−1^) and lowest in cushion vegetation (1.16 g·kg^−1^; Figure 3c).

### 2.3. Homeostasis of Plant Functional Groups

In alpine meadow and alpine cushion vegetation, the homeostasis models of C, N, and P stoichiometric traits for Gramineae, Cyperaceae, and forbs were not statistically significant (*p* > 0.05) (Table 1). In contrast, within alpine shrubland, Cyperaceae showed weak sensitivity to phosphorus (H^−1^ = 0.5526) (*p* < 0.05) and weak homeostasis in the C:P ratio (H^−1^ = 0.4669) (*p* < 0.05). These findings indicate that Cyperaceae in alpine shrubland exhibit greater variability in P accumulation, underscoring their sensitivity to phosphorus limitation along the zonal vegetation gradient.

### 2.4. Environmental Drivers of Stoichiometric Variation in Functional Groups

**Figure 4:**
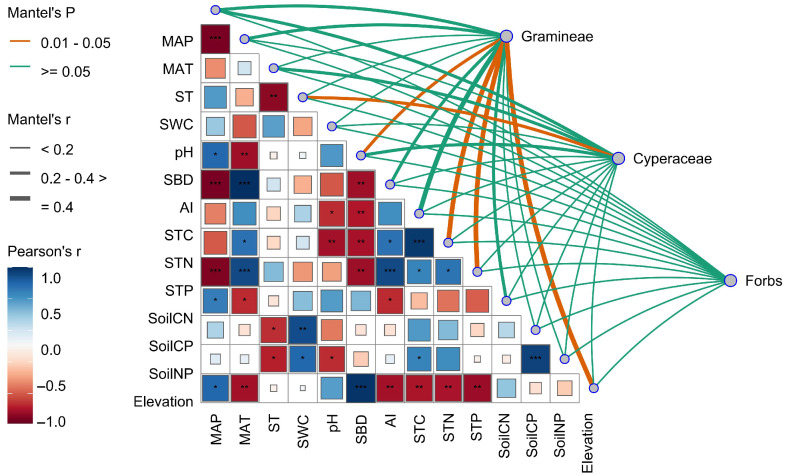
Mantel tests between plant stoichiometric traits (carbon, nitrogen, phosphorus contents and ratios) of each functional group and environmental variables. MAT, mean annual temperature; MAP, mean annual precipitation; ST, soil temperature; SWC, soil water content; SBD, soil bulk density; AI, aridity index; STC, soil total carbon; STN, soil total nitrogen; STP, soil total phosphorus; CN, C:N ratio; CP, C:P ratio; NP; N:P ratio. * indicates *p* < 0.05; ** indicates *p* < 0.01; *** indicates *p* < 0.001.

Mantel tests revealed that elevation was positively correlated with mean annual precipitation (MAP) and significantly negatively correlated with mean annual temperature (MAT) and soil nutrient content. The stoichiometric traits of Gramineae were significantly correlated with soil bulk density (SBD), soil total nitrogen (STN), soil total phosphorus (STP), and elevation (*p* < 0.05). Cyperaceae stoichiometry was significantly associated with soil water content (SWC) (*p* < 0.05), while no significant relationships were detected between forb stoichiometry and any environmental factors (Figure 4). Overall, elevation acts as an integrative geographic factor that indirectly shapes the spatial variation in plant C, N, and P stoichiometric traits by modulating both climatic and edaphic conditions.

**Figure 5:**
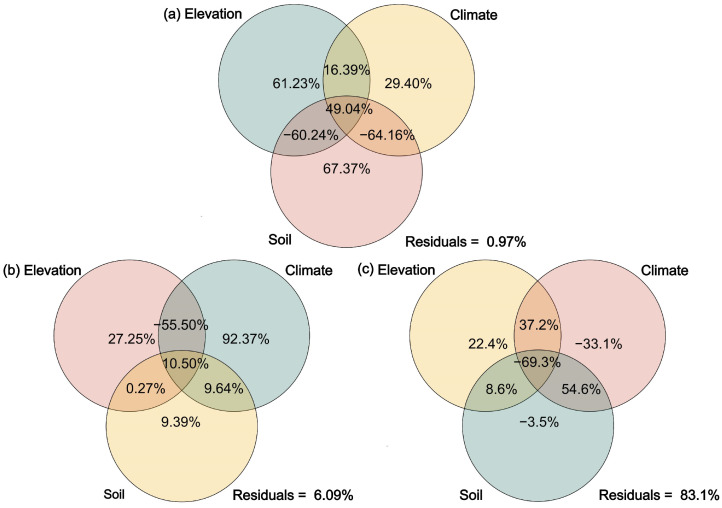
Variance partitioning of the effects of elevation, climate, and soil variables on the stoichiometric traits of different plant functional groups: (**a**), Gramineae plants; (**b**), Cyperaceae plants; (**c**), forbs.

Variance partitioning results showed that elevation, climate, and soil factors independently explained 61.23%, 29.4%, and 67.37% of the variation in stoichiometric traits of Gramineae (Figure 5a). For Cyperaceae, the independent explanatory power of elevation, climate, and soil factors was 27.25%, 92.37%, and 9.39% (Figure 5b). In the case of forbs, only elevation independently explained a significant portion (22.4%) of the variation (Figure 5c). Overall, Gramineae stoichiometric traits were primarily driven by soil nutrients and related edaphic conditions; Cyperaceae were mainly regulated by climate, especially water availability, while forbs were strongly associated with elevation as an indirect environmental driver.

**Figure 6:**
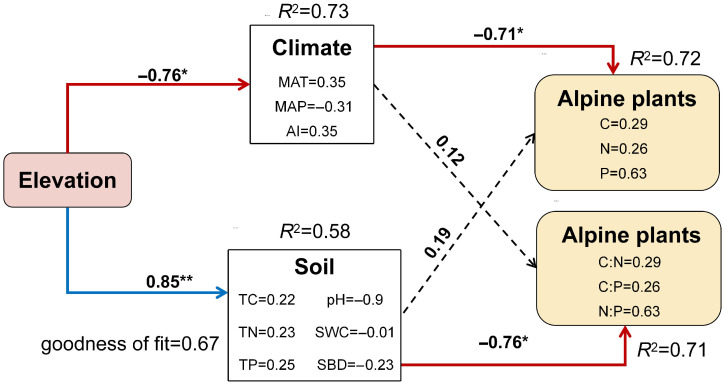
Structural equation model showing the effects of environmental factors on plant stoichiometric traits. MAT, mean annual temperature; MAP, mean annual precipitation; AI, aridity index; TC, total carbon; TN, total nitrogen; TP, total phosphorus; SWC, soil water content; SBD, soil bulk density; C, carbon; N, nitrogen; P, phosphorus. * indicates *p* < 0.05; ** indicates *p* < 0.01.

Results from the structural equation model (Figure 6) further revealed that elevation influenced plant C, N, and P stoichiometry mainly through alterations in soil physicochemical properties (path coefficient = 0.85) and climate conditions (path coefficient = −0.76). Specifically, climate had a strong direct effect on plant C, N, and P contents (path coefficient = −0.71), whereas soil conditions primarily affected the stoichiometric ratios of C:N:P (path coefficient = −0.76). In summary, elevation indirectly affects plant stoichiometry by modulating climate and soil properties. Both climate and soil act as direct environmental drivers, exerting significant negative effects on plant stoichiometric traits.

## 3. Discussion

By comparing the stoichiometric characteristics and homeostasis of different plant functional groups across vegetation types, we found that in alpine shrubland, where soil N is abundant (Figure 1), the N content and N:P ratio of Gramineae plants and forbs were highest, while Cyperaceae plants had the lowest N:P ratio but the highest P content. Functional groups exhibited stoichiometric traits distinct from those in the other two vegetation types (Figure 3), thus supporting our first hypothesis. However, stoichiometric homeostasis results (Table 1) showed that only Cyperaceae plants in alpine shrubland exhibited weak sensitivity in P (H^−1^ = 0.5526) and weak homeostasis in C:P (H^−1^ = 0.4669). Therefore, our second hypothesis was rejected, as Cyperaceae plants showed greater sensitivity to climate change, with mean annual temperature, annual precipitation, and soil moisture significantly affecting their C:N:P ratios (Figure 4 and Figure 5b). In contrast, forbs, composed of diverse and widely distributed species, showed high ecological adaptability and strong stoichiometric homeostasis. Our findings provide important theoretical insights into the mechanisms of ecological adaptation and community assembly in alpine ecosystems under the context of ongoing climate warming.

### 3.1. Stoichiometric Traits of Plant Functional Groups Across Altitudinal Vegetation Zones

Due to the mountain effect, an inversion layer and a precipitation belt developed in the central part of the study area [26,27], allowing alpine shrubland to form a transitional vegetation zone between alpine meadow and cushion vegetation. Our findings reveal that both the stoichiometric traits and homeostasis patterns of plant functional groups in this transitional zone (alpine shrubland) differ significantly (*p* < 0.05) from those in the adjacent vegetation types, highlighting distinct adaptive strategies in response to heterogeneous environmental conditions and resource availability. Specifically, N content in Poaceae (Gramineae) and forb plants were significantly higher in alpine shrubland (Figure 3b), accompanied by the lowest C:N ratios (Figure 3d), suggesting greater N uptake, potentially enhancing protein synthesis and growth. However, their C:P and N:P ratios were highest (Figure 3e,f), possibly indicating intensified P limitation or a rapid response to N enrichment [1]. In contrast, Cyperaceae exhibited a distinct pattern, with the highest TP concentration in alpine shrubland (Figure 3c) and the lowest C:P and N:P ratios, indicating a strong capacity for P accumulation or a strategy to maintain nutrient balance in a P-rich but N-limited environment [28].

Our study shows that P in Cyperaceae in alpine shrubland exhibited weak sensitivity (H^−1^ = 0.5526), and its C:P ratio displayed weak homeostasis (H^−1^ = 0.4669) (Table 1). This suggests that in transitional habitats, Cyperaceae may have more flexible nutrient acquisition and allocation mechanisms in response to environmental variation. Such reduced homeostasis may result from complex below-ground nutrient dynamics driven by rhizospheric competition, litter inputs, and microsite variation in water and temperature [29]. For Cyperaceae, the weak sensitivity of P and weak homeostasis of C:P likely reflect a highly adaptive nutrient use strategy, where plants adjust nutrient uptake or internal allocation to enhance competitiveness under heterogeneous resource conditions [30].

Furthermore, ecotones often harbor the greatest overlap in functional diversity and ecosystem processes, where community structural heterogeneity and interspecific functional redundancy may obscure nutrient response signals, resulting in apparent reductions in homeostatic regulation [31]. As a result, ecosystem functioning in transitional zones may increasingly depend on a few environmentally responsive taxa—such as Cyperaceae in our study. In summary, ecotones not only serve as critical boundaries for plant turnover and vegetation shifts but also reflect divergent stoichiometric strategies and resource use plasticity among plant functional groups in response to environmental gradients. These differences may drive vegetation succession in alpine ecosystems under future climate scenarios, particularly via shifts in P availability and subsequent impacts on Cyperaceae composition.

### 3.2. Environmental Response Strategies of Different Plant Functional Groups

The C:N ratio of plants not only indicates nitrogen use efficiency but also reflects nutrient use strategies under varying resource conditions [32]. According to Grime’s CSR theory, plants can be categorized as competitors (C), stress-tolerators (S), or ruderals (R), with low C:N associated with competitive plants and high C:N with conservative strategies [16]. In this study, vegetation types from alpine cushion vegetation to shrubland to meadow reflect a successional trajectory from early to late stages along the altitudinal gradient. Poaceae (Gramineae) and forbs exhibited a V-shaped trend in their C:N ratios, with the lowest values in the mid-successional alpine shrubland (Figure 3d), indicating a more acquisitive, competition-oriented strategy during this phase. This is likely due to the higher total N content in shrubland soils (Figure 1b), which facilitates coexistence of fast-growing plants [33]. Thus, N-rich conditions in shrubland likely support rapid N uptake and reduced C:N in Poaceae (Gramineae) and forbs, indicating resource-driven strategy shifts during succession.

The carbon-to-phosphorus (C:P) ratio mainly reflects plant growth rates, with lower values associated with fast-growing species that require more ribosomal RNA [34]. In our results, Poaceae (Gramineae) had the lowest C:P in meadow, while Cyperaceae and forbs showed the lowest C:P in shrubland and cushion vegetation, respectively (Figure 3e). This suggests different groups function as pioneer plants at different successional stages [35]. For example, Poaceae (Gramineae) may dominate early meadow stages, while Cyperaceae and forbs contribute to community establishment in shrubland and cushion vegetation, respectively. Therefore, increased soil P availability under warming and moistening climates [36] may preferentially alter Poaceae (Gramineae) and Cyperaceae stoichiometry, driving plant replacement and vegetation transitions along the alpine gradient.

Soil N:P values in our study area were consistently lower than the global grassland average of 14 [28], indicating widespread N limitation typical of early succession or nutrient-poor alpine ecosystems. Among vegetation types, shrubland had significantly higher soil N:P ratios than meadow and cushion vegetation (Figure 1f), suggesting improved nutrient supply during the mid-successional stage. Although plant N:P ratios often exhibit synchronicity with soil N:P [37], Cyperaceae showed the lowest plant N:P in shrubland despite elevated soil N:P (Figure 3f), indicating a decoupling and high plasticity in nutrient acquisition [38]. This flexibility may be attributed to rhizospheric regulation, microbial associations, and diverse nutrient uptake strategies [39].

Overall, C:N:P ratio shifts across the altitudinal gradient reflect the divergent ecological responses of plant functional groups to changing resources and stressors during vegetation succession. By modulating stoichiometric ratios, different groups adapt to specific successional niches, reinforcing the importance of nutrient coupling between plants and soils in maintaining alpine ecosystem stability.

### 3.3. Drivers of Stoichiometric Variation in Alpine Grassland Plants

Variance partitioning and correlation analyses revealed that stoichiometric variation in Poaceae (Gramineae) was primarily explained by soil factors, especially bulk density and soil N:P ratio (Figure 4 and Figure 5a), suggesting high sensitivity to soil structure and nutrient availability [40]. In contrast, Cyperaceae responded most strongly to climatic variables—mean annual temperature, precipitation, and soil water content significantly influenced its C:N:P ratios (Figure 4 and Figure 5b), indicating strong ecological plasticity under changing hydrothermal conditions [10]. Forbs were mainly influenced by elevation (Figure 5c), reflecting broad ecological adaptability to complex stresses such as low temperature and high radiation [41].

These divergent drivers likely stem from differing functional traits: Poaceae (Gramineae), with high growth rates and nutrient demands, are more dependent on direct soil nutrient supply (Figure 6) [28]. Cyperaceae, including plants like *Kobresia pygmaea*, show strong genetic and physiological responses to cold, including increased expression of stress-resistance genes and enhanced energy metabolism under low temperatures [42], making them particularly responsive to climate. In contrast, forbs encompass diverse plants with wide ecological niches and broad distributions [43]. Elevation, as an integrative factor reflecting climatic and edaphic gradients, may promote their nutrient use efficiency and ecological plasticity [27]. In conclusion, the C:N:P ratios of different functional groups reflect their adaptive strategies to multi-factorial environmental gradients. Through stoichiometric adjustments, these groups modulate their responses to resource heterogeneity and climatic stress, thereby contributing to the structural and functional stability of alpine ecosystems.

## 4. Materials and Methods

### 4.1. Study Area

**Figure 7:**
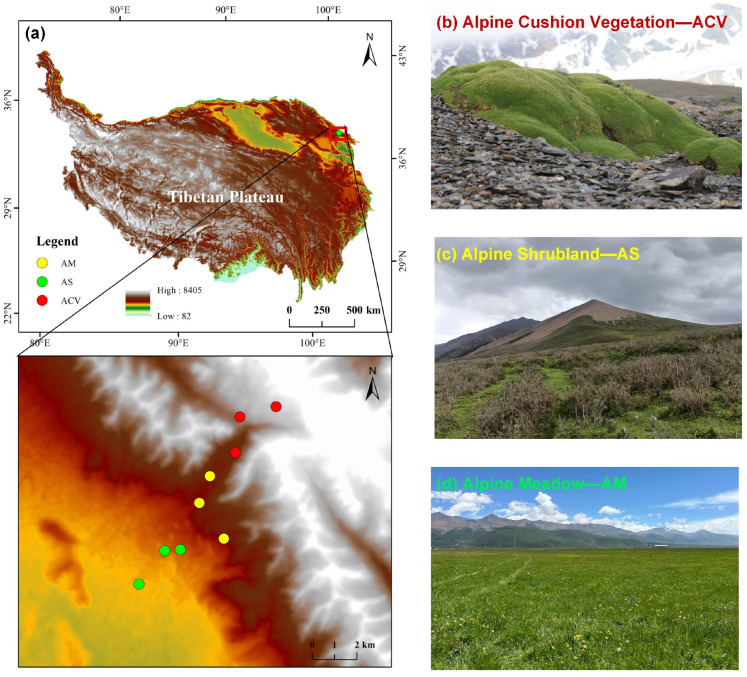
Overview of the study area. (**a**) Study location; (**b**–**d**) represent the vegetation types analyzed in this study.

The study area is located on Gangshika Peak, in the eastern section of the Qilian Mountains (37°37′42.20″ N–37°42′1.56″ N, 101°24′9.37″ E–101°28′11.76″ E), with an average elevation of 3800 m and a highest point of 5254 m. According to the Köppen–Geiger climate classification, the study area falls into the ET (alpine tundra climate) type, characterized by cold conditions, short, cool summers, and long, severe winters [44]. The mean annual temperature is −1.7 °C, and the mean annual precipitation is 599 mm, with 70–80% of the precipitation concentrated during the plant growing season (May to September). The dominant vegetation types include the following: alpine meadow (Figure 7a), dominated by *Carex alatauensis* and *Elymus nutans*; alpine shrubland (Figure 7b), dominated by *Dasiphora fruticosa*, *Caragana jubata*, and *Salix oritrepha*; and alpine cushion vegetation (Figure 7c), dominated by *Thylacospermum caespitosum*, *Sibbaldia tetrandra*, and *Arenaria kansuensis* (all scientific names follow Plants of the World Online [45]). The soil types correspond to the vegetation types and include alpine meadow soil, alpine shrub meadow soil, and alpine desert soil, according to the Chinese Soil Taxonomy [46]. Based on the World Reference Base for Soil Resources, these soils are classified mainly as Cambisols and Leptosols [47]. Detailed information on plot characteristics and soil physicochemical properties is provided in Table 2 and Table 3.

### 4.2. Experimental Design

In August 2024, three typical vertical vegetation types were selected along an altitudinal gradient in the study area: alpine meadow, alpine shrubland, and alpine cushion vegetation. For each vegetation type, three plots (50 m × 50 m) were established (see Figure 7, Table 2), totaling nine plots. Within each plot, five subplots were arranged using a quincunx (five-point) sampling design, resulting in 45 subplots in total. The subplot size was 1 m × 1 m for alpine meadow and 5 m × 5 m for alpine shrubland and alpine cushion vegetation. In each subplot, plant name, individual count, plant height above ground, and canopy cover were recorded. Above-ground biomass of each species was harvested at ground level, brought to the laboratory, pre-dried at 105 °C for 30 min to inactivate enzymatic activity, oven-dried at 65 °C for 48 h, ground using a ball mill, and sieved through a 100-mesh screen for C, N, and P contents analysis. Following plant sampling, soil bulk density was measured using a 100 cm^3^ soil ring sampler in each subplot. Additionally, five soil cores (0–20 cm depth) were randomly taken per subplot, composited, and passed through a 2 mm sieve to remove roots, litter, and gravel. The mixed samples were transported to the laboratory for analysis of soil pH, TC, TN, and TP contents.

### 4.3. Environmental Measurements and Sample Determinations

In the field, a portable automatic weather station (HOBO H21-USB, Onset, Bourne, MA, USA) was used to continuously monitor soil temperature and moisture at a depth of 0–20 cm. Soil pH was determined using a pH meter after mixing soil with deionized water at a 2.5:1 water-to-soil ratio [48]. Carbon and N contents of both soil and plant samples were measured using an elemental analyzer (Vario EL III, Elementar, Langenselbold, Germany), while total P was determined using the molybdenum–antimony–scandium colorimetric method [49]. Monthly gridded precipitation data (1 km resolution, 1901–2024) were obtained from the National Earth System Science Data Center [50], and temperature and precipitation data were also retrieved from the China Meteorological Data Service Center [51]. Spatial interpolation was conducted using Anusplin 4.2 software to generate raster-based climate datasets. Meteorological data corresponding to each sampling plot were extracted from the gridded datasets using ArcGIS 10.7. Extraterrestrial radiation data used in this study were provided by the National Tibetan Plateau Data Center [52].

The aridity index (AI) was calculated for each plot based on potential evapotranspiration and mean annual precipitation (Equations (1) and (2)) [53,54].

(1) $$PET=0.0023\times (T_{mean}+17.8)\times {\left(T_{max}-T_{min}\right)}^{0.5}\times R_{a}$$

(2) $$AI=\frac{MAP}{PET}$$

where T_mean_ is the mean annual temperature, T_max_ is the annual maximum temperature, T_min_ is the annual minimum temperature, and R_a_ is the extraterrestrial radiation. MAP is the mean annual precipitation, and PET is the potential evapotranspiration at each sampling site.

The homeostasis of plant stoichiometric traits was assessed by analyzing the relationship between elemental content in soils and plants, and their respective stoichiometric ratios (Equation (3)). The homeostasis index (H) was calculated following Persson et al. (2012) using the following equation [55]:

(3) $$H^{-1}=\left[lg\left(y\right)-lg(c)\right]/lg(x)$$

where x and y represent the stoichiometric traits of soil and plant tissues, respectively, and c is the fitted constant. The absolute value of H^−1^ is commonly used as an indicator of stoichiometric homeostasis strength. When the regression equation is statistically significant (*p* < 0.05), the classification of homeostasis is as follows:

∣H^−1^∣ ≤ 0.25: absolute homeostatic

0.25 < ∣H^−1^∣ ≤ 0.5: weakly homeostatic

0.5 < ∣H^−1^∣ ≤ 0.75: weakly sensitive

∣H^−1^∣ > 0.75: sensitive

### 4.4. Statistical Analyses

Data were organized using Microsoft Excel 2019 (Microsoft Corporation, Redmond, WA, USA). One-way analysis of variance (ANOVA) was performed in SPSS 27 (IBM Corporation, Armonk, NY, USA) after standardizing the data, to compare the differences in stoichiometric traits of different plant functional groups across vegetation types, with a significance level of α = 0.05. Mantel tests were conducted in R Studio 4.3.2 using the dplyr and linkET packages to examine the correlations between C, N [56], and P stoichiometry of plant functional groups and environmental factors including climate and soil physicochemical properties. Redundancy analysis (RDA) was performed in Canoco 5 to explain the overall variation in stoichiometric traits in relation to environmental variables. Variance partitioning and structural equation modeling (SEM) were carried out in R version 4.3.2 (R Core Team, Vienna, Austria) using the vegan and plspm packages [57,58], respectively, to quantify the contributions of climatic and edaphic factors to stoichiometric variation, and to explore the pathways through which environmental variables influence plant stoichiometry along the elevational gradient.

To avoid pseudo-replication, we treated plots (*n* = 9) as independent replicates, with vegetation type as a fixed effect and plot as a random intercept, fitted using linear/generalized linear mixed-effects models (Satterthwaite approximation). We repeated all key analyses on plot-level means (*n* = 9) and conducted leave-one-plot-out sensitivity checks. Spatial autocorrelation of residuals was assessed with Moran’s I. We reported partial R^2^/η^2^ and Cohen’s d with 95% CIs, and complemented *p*-values with permutation/bootstrap-based inference.

## 5. Conclusions

As an ecological ecotone, alpine shrubland exhibit enhanced plant accumulation of C and N content. At the functional group level, the stoichiometric traits of Poaceae (Gramineae) plants are primarily regulated by soil structure and soil nitrogen and P availability, while Cyperaceae plants are more sensitive to hydrothermal conditions. In general, stoichiometric traits of alpine grassland plants tend to be stable, with both climate and soil factors exerting strong negative effects on elemental accumulation and stoichiometric ratios. This suggests that under harsh climatic conditions and nutrient-poor soils, plants tend to adopt tolerant strategies characterized by increased accumulation of N and P nutrients. Notably, Cyperaceae plants in alpine shrubland showed weak sensitivity and low homeostasis for P and C:P ratio, indicating that P supply and availability play a critical regulatory role in shaping the fitness of Cyperaceae plants, and may even determine the composition and distribution of plant functional groups along altitudinal gradients. In summary, plant responses to environmental changes along the alpine altitudinal spectrum are not merely passive. Instead, plants actively adapt to resource limitations through differentiation among functional groups and adjustments in elemental homeostasis. These strategies highlight the adaptive plasticity and regulatory capacity of alpine plants in coping with environmental heterogeneity. Our findings provide important theoretical insights into the mechanisms of ecological adaptation and community assembly in alpine ecosystems under the context of ongoing climate warming.

## Figures and Tables

**Table 1:** Stoichiometric homeostasis of carbon (C), nitrogen (N), and phosphorus (P) in plant functional groups under different vegetation types.

Lg(x)	Lg(y)	Functional Group	AM		AS		ACV	
			*p*	H^−1^	*p*	H^−1^	*p*	H^−1^
C_soil_	C_plant_	Gra	0.224	0.6363	0.431	0.8291	0.474	0.3393
		Cyp	0.250	3.5840	0.765	0.0611	0.468	0.2606
		Forbs	0.137	1.9380	0.449	0.7253	0.961	0.0148
N_soil_	N_plant_	Gra	0.549	3.6400	0.958	0.0433	0.480	3.8840
		Cyp	0.590	2.5110	0.873	0.1254	0.361	2.2430
		Forbs	0.736	2.2090	0.079	0.4085	0.504	2.5940
P_soil_	P_plant_	Gra	0.173	0.9155	0.283	1.0240	0.187	0.1243
		Cyp	0.669	0.6885	0.026 *	**0.5526**	0.322	0.4667
		Forbs	0.107	0.5235	0.767	0.3049	0.686	0.0627
C_soil_:N_soil_	C_plant_:N_plant_	Gra	0.270	4.2270	0.988	0.0110	0.446	6.3700
		Cyp	0.075	2.8380	0.908	0.0879	0.297	3.4280
		Forbs	0.928	0.4615	0.093	0.4598	0.423	4.8640
C_soil_:P_soil_	C_plant_:P_plant_	Gra	0.213	1.2840	0.290	0.7289	0.107	0.2193
		Cyp	0.282	1.7440	0.002 **	**0.4669**	0.561	0.2812
		Forbs	0.088	1.1360	0.575	0.4886	0.295	0.1270
N_soil_:P_soil_	N_plant_:P_plant_	Gra	0.173	3.2710	0.411	0.6936	0.207	0.8989
		Cyp	0.290	2.9550	0.549	0.7550	0.947	0.0431
		Forbs	0.433	2.3870	0.470	0.6913	0.267	0.6203

AM, alpine meadow; AS, alpine shrubland; ACV, alpine cushion vegetation; Gra, gramineae; Cyp, cyperaceae; H, homeostasis index; *p*, *p* value, * indicate *p* < 0.05, ** indicates *p* < 0.01.

**Table 2:** Vegetation composition and geographic–topographic information (coordinates, elevation, and slope) for each study plot.

Vegetation Type	Dominant Species	Plot ID	Elevation (m)	Geographic Coordinates	Slope (°)
Alpine Meadow	*Carex alatauensis*, *Elymus nutans*	1	3283	E 101°24′9.37″ N 37°37′42.20″	0
		2	3361	E 101°24′55.10″ N 37°38′33.62″	0
		3	3410	E 101°25′23.86″ N 37°38′51.23″	11
Alpine Shrubland	*Dasiphora fruticosa*, *Caragana jubata*, *Salix oritrepha*	4	3504	E 101°28′43.50″ N 37°38′51.23″	18
		5	3600	E 101°25′56.03″ N 37°39′40.70″	15
		6	3666	E 101°26′13.62″ N 37°40′19.35″	25
Alpine Cushion Vegetation	*Thylacospermum caespitosum*, *Sibbaldia tetrandra*	7	3868	E 101°26′59.5″ N 37°40′54.12″	36
		8	4104	E 101°27′5.33″ N 37°41′44.95″	9
		9	4371	E 101°28′11.76″ N 37°42′1.56″	17

**Table 3:** Soil physicochemical properties (pH, temperature, water content, bulk density) for each vegetation type.

Vegetation Type	pH	Temperature (°C)	Water Content (%)	Bulk Density (g·cm^−3^)
Alpine Meadow	6.51 ± 0.02 a	14.78 ± 0.03 a	15.04 ± 0.01 a	0.58 ± 0.01 a
Alpine Shrubland	6.23 ± 0.37 a	11.02 ± 0.89 c	33.19 ± 0.02 a	0.83 ± 0.04 a
Alpine Cushion Vegetation	6.91 ± 0.15 a	13.07 ± 1.57 b	21.90 ± 0.05 b	1.37 ± 0.21 b

Different lowercase letters indicate significant differences among vegetation types.

## Data Availability

The data presented in this study are available on request from the corresponding author. The data are not publicly available due to project restrictions.
